# High-Intensity Interval Training Is Effective at Increasing Exercise Endurance Capacity and Is Well Tolerated by Adults with Cystic Fibrosis

**DOI:** 10.3390/jcm9103098

**Published:** 2020-09-25

**Authors:** Abbey Sawyer, Vinicius Cavalheri, Sue Jenkins, Jamie Wood, Nola Cecins, Natasha Bear, Bhajan Singh, Daniel Gucciardi, Kylie Hill

**Affiliations:** 1 School of Physiotherapy and Exercise Science, Faculty of Health Science, Curtin University, Kent Street, Bentley, WA 6102, Australia; abbey.sawyer@live.com.au (A.S.); vinicius.cavalheri@curtin.edu.au (V.C.); s.jenkins@curtin.edu.au (S.J.); Jamie.Wood@health.wa.gov.au (J.W.); D.Gucciardi@curtin.edu.au (D.G.); 2 Physiotherapy Department, Sir Charles Gairdner Hospital, Perth, WA 6009, Australia; Nola.Cecins@health.wa.gov.au; 3 Institute for Respiratory Health, Perth, WA 6009, Australia; 4 Allied Health, South Metropolitan Health Service, Perth, WA 6150, Australia; 5 Institute of Health Research, Fremantle Campus, University of Notre Dame Australia, Fremantle, WA 6160, Australia; Natasha@bearstats.com.au; 6 West Australian Sleep Disorders Research Institute, Nedlands, WA 6009, Australia; Bhajan.Singh@health.wa.gov.au; 7 Department of Pulmonary Physiology and Sleep Medicine, Sir Charles Gairdner Hospital, Perth, WA 6009, Australia; 8 Faculty of Science, University of Western Australia, Crawley, WA 6009, Australia

**Keywords:** cystic fibrosis, exercise, high intensity interval training

## Abstract

Background: To optimize outcomes in people with cystic fibrosis (CF), guidelines recommend 30 to 60 min of moderate-intensity aerobic exercise on most days. Accumulating this volume of exercise contributes importantly to the substantial treatment burden associated with CF. Therefore, the main aim of this study was to investigate the effects of low-volume high-intensity interval training (HIIT) on exercise capacity in people with CF. Methods: This randomized controlled trial included people with CF aged ≥15 years, who were allocated to either eight weeks of thrice-weekly 10-min sessions of HIIT (experimental group) or eight weeks of weekly contact (control group). Before and after the intervention period, participants completed measurements of time to symptom limitation (T_lim_) during a constant work rate cycle ergometry test (primary outcome), and maximal work rate (W_max_) during a ramp-based cycle ergometry test and health-related quality of life (HRQoL). Results: Fourteen participants (median (IQR) age 31 (28, 35) years, forced expiratory volume in 1 second (FEV_1_) 61 (45, 80) % predicted) were included (seven in each group). Compared to the control group, participants in the experimental group demonstrated a greater magnitude of change in T_lim_, W_max_ (*p* = 0.017 for both) and in the physical function domain of HRQoL (*p* = 0.03). No other between-group differences were demonstrated. Mild post-exercise muscle soreness was reported on a single occasion by four participants. Overall, participants attended 93% of all HIIT sessions. Discussion: Eight weeks of low-volume (i.e., 30-min/week) HIIT produced gains in exercise capacity and self-reported physical function and was well tolerated by people with CF.

## 1. Background

People with cystic fibrosis (CF) have reduced exercise capacity [1]. The cause of such reduction is multifactorial and includes impairments in lung function as well as peripheral and respiratory muscle deconditioning, the latter of which contributes to breathlessness and muscle fatigue during exercise [2,3]. In this population, as greater exercise capacity has been associated with better health-related quality of life (HRQoL) [4,5] and survival [6,7,8], maintaining or improving exercise capacity is an important treatment goal.

Exercise capacity can be optimized by undertaking ‘traditional’ continuous, moderate intensity, aerobic exercise training [9,10,11,12,13,14]. The Australian and New Zealand CF clinical practice guidelines recommend moderate-intensity exercise on most days of the week [14]. However, this type of exercise training is time consuming, adding to the already high daily treatment burden, and may not be feasible due to a ‘lack of time’ [15]. Further, the intensity achieved during long periods of continuous training is likely to be constrained by intolerable symptoms [16]. An alternative training approach, such as low-volume high intensity interval training (HIIT), may be a more achievable and efficient method to optimize exercise capacity.

High-intensity interval training consists of short periods of ‘work’, interrupted by periods of ‘rest’ [17]. The ‘rest’ period allows for partial recovery of symptoms of breathlessness and muscle fatigue, and therefore offers the opportunity to optimize the training intensity achieved during the ‘work’ periods [18,19]. In healthy adults [19] and people with chronic obstructive pulmonary disease (COPD) [20], HIIT is well tolerated and offers similar gains in exercise capacity to continuous training, with less intense symptoms [21,22], and lower training time [23] or work [24] per session. Preliminary data suggest that HIIT is feasible and well-tolerated in people with CF [16]. However, the effectiveness has not been investigated in a randomized controlled trial (RCT).

The primary research question of this RCT is: In people with CF, what is the effect of an eight-week low-volume HIIT program, compared with weekly contact and no formal exercise training, on exercise capacity (primary outcome), HRQoL, exercise self-efficacy, feelings of anxiety and depression, and exercise enjoyment (secondary outcomes)?

Secondary research questions are: In participants allocated to the experimental group (HIIT), (i) what proportion develop post-exercise muscle soreness each week during the eight-week HIIT program, and how severe is this symptom? (ii) how well do the participants tolerate the program? (iii) what are the participants’ reflections on the ‘facilitators’ and ‘barriers’ following the program? and (iv) which behaviour change techniques (BCTs) are organically employed to optimize participation in the HIIT intervention?

## 2. Methods

### Study Design

**Figure 1:**
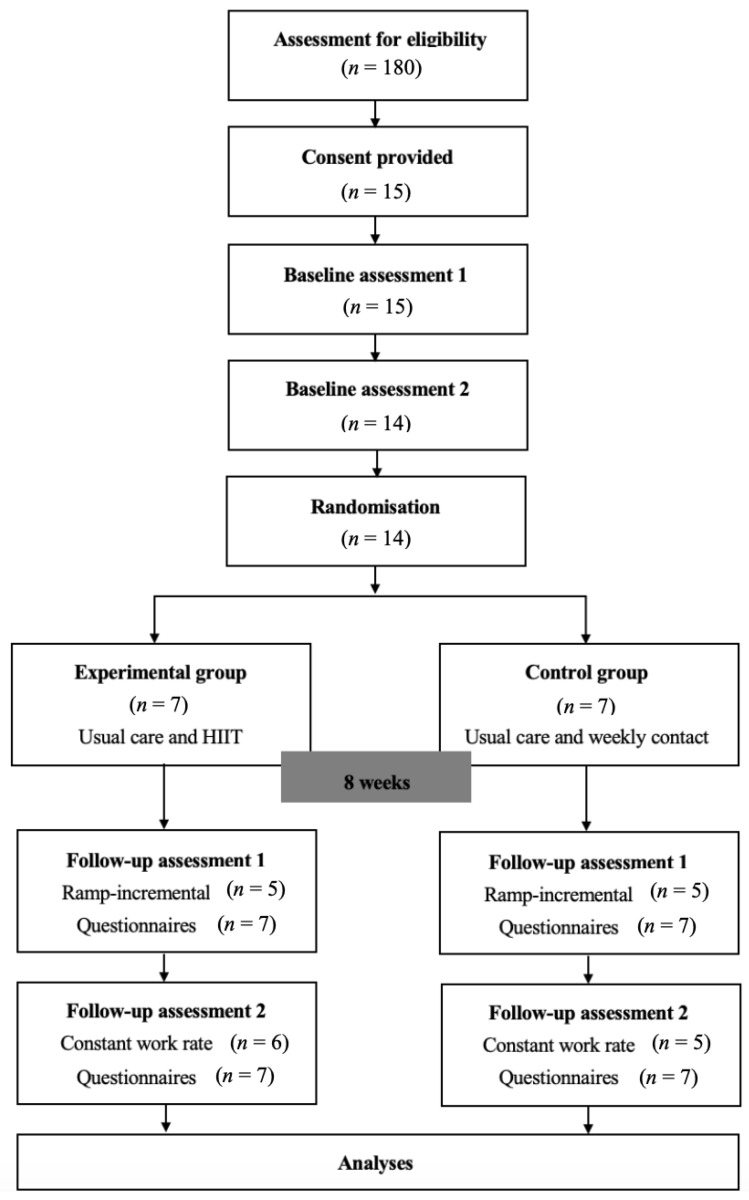
Study flow diagram. Notes: During the first baseline and follow-up assessments, the maximal incremental ramp-based cycle ergometry test was performed. During the second baseline and follow-up assessment, the constant work rate cycle ergometry test was performed. Questionnaires were completed across all assessment sessions. There were 180 people under the care of the CF center at Sir Charles Gairdner Hospital. Forty-one (23%) were ineligible to participate as they either lived outside the Perth metropolitan area (*n* = 40) or were unable to read and understand English (*n* = 1). Of the remaining participants (*n* = 139), 124 declined to participate. Reasons cited for non-participation included: not willing to travel to hospital locations (*n* = 59); ongoing medical issues limiting participation (*n* = 29); already exercising at least twice per week (*n* = 14); and being time-poor, or having a work schedule that did not permit participation (*n* = 7). Four potential participants declined without a specific reason. A further nine potential participants expressed interest in the study, but did not respond to subsequent contact attempts. Two potential participants who were colonized with nontuberculous mycobacteria expressed interest in the study, but were not permitted to take part due to the risk of cross-infection in the exercise laboratory. HIIT: high-intensity interval training.

This study was a prospective single-blinded (outcome assessor) RCT [25]. Data collection took place between October 2017 and October 2019. Figure 1 shows participant flow through the RCT. The registration number is 12617001271392 (ANZCTR).

## 3. Participants

### 3.1. Recruitment and Consent

Potential participants were identified at scheduled outpatient clinic appointments and provided with an information sheet summarizing the study. They were contacted (via telephone) within 48 h of their clinic visit to discuss their willingness to participate. Prior to data collection, written informed consent was obtained from all participants. This study was approved by the Human Research Ethics Committee at Sir Charles Gairdner Hospital, on behalf of Sir Charles Gairdner Hospital and Perth Children’s Hospital (RGS 0000000065) with reciprocal approval from Curtin University (HRE2017-0651).

### 3.2. Eligibility Criteria

Individuals were eligible to participate in the study if they were aged ≥ 15 years and had a body mass index > 16 kg·m^−2^. Exclusion criteria comprised: (i) recent (within the previous four weeks) exacerbation of CF which required oral or intravenous antibiotics; (ii) a comorbidity that would impact on their ability to undertake a maximal incremental ramp-based cycle ergometry test; (iii) poorly controlled diabetes; (iv) previous or current listing for lung transplantation; (v) participation in structured exercise at a moderate intensity two or more times per week for the previous three months; and (vi) inability to provide written informed consent.

## 4. Intervention Period

### 4.1. Experimental Group

The intervention was conducted and reported in accordance with the Template for Intervention Description and Replication [26] and Consensus on Exercise Reporting Template [27,28]. Each training session involved a 2 min ‘warm up’, followed by a 30 s ‘work’ phase and 30 s ‘rest’ period, repeated six times. The work to rest periods were followed by a 2 min ‘cool down’ period. Therefore, the total training time per session was 10 min. Each session was overseen by a physiotherapist who was trained in the management of people with CF. The training program commenced with a ‘lead in’ phase which involved only two sessions of HIIT in weeks 1 and 2. Between weeks 3 and 8, HIIT was undertaken three times a week (i.e., total 22 sessions over 8 weeks). The training intensity was prescribed using measurements of maximum work rate (W_max_) achieved during the ramp-based cycle ergometry test completed prior to randomization. Specifically, the first training session was prescribed at 60% of W_max_, with the goal of achieving a training intensity equal to 80% of W_max_ by the end of week 2. Thereafter, training intensity was increased as rapidly as symptoms of breathlessness and muscle fatigue permitted. Training was completed at one of two sites, with participants permitted to choose the site and time that was most convenient. The model of exercise bike used for the HIIT sessions was identical across sites (Orbit Eco Generator Interval Bike OEB2002, Perth, Australia). Participants’ observations (pulse rate (PR) and oxygen saturation (SpO_2_)) were continuously monitored using a Masimo Rad v5 oximeter (Masimo SET Rad-5, Masimo, California, USA). Further detail on the BCTs included within the HIIT intervention are available in Table S1.

If a participant in either group reported the onset of symptoms indicative of a respiratory exacerbation [29], they were referred to the CF team for medical review. Those allocated to the experimental group were invited to continue participating in HIIT sessions once deemed to be medically stable by a CF clinician. In the event of missed attendance, the training program was extended by up to two weeks.

### 4.2. Control Group

Participants allocated to the control group were contacted once a week by a preferred method (phone, email, or SMS) to discuss changes to their symptoms, healthcare utilization, and participation in exercise over the preceding week.

### 4.3. Assessment Periods

Both prior to randomization (i.e., baseline) and following the intervention period (i.e., follow-up), participants completed assessments over two non-consecutive days. Each assessment lasted under 1.5 h, and both visits were completed within two weeks. In order to evaluate the effect of the experimental intervention (i.e., HIIT), measures of exercise capacity, HRQoL, exercise self-efficacy, feelings of anxiety, depression, and exercise enjoyment were collected in both groups. The follow-up assessment of these outcomes was completed by an assessor who was blinded to group allocation. In the experimental group only, post-exercise muscle soreness (measured weekly), tolerance (measured at every HIIT session), and participant debriefs (post-HIIT program) were assessed. High-intensity interval training sessions were audio recorded for reporting of BCTs and fidelity checking.

## 5. Measurements Related to the Primary Research Question

### 5.1. Primary Outcome

#### Exercise Capacity

Exercise capacity was measured using two exercise tests, using a protocol which is reported elsewhere [25]. All equipment was calibrated at time intervals in accordance with the manufacturer’s recommendations. In order to establish the W_max_ in each participant, a ramp-based cycle ergometry test was undertaken on an electronically-braked cycle ergometer (Ergoselect 100; Ergoline, Bitz, Germany). Thereafter, on a non-consecutive day, endurance exercise capacity was assessed using a constant work rate test [30]. This test comprised 1 min of rest, 1 min of unloaded cycling, after which the work rate was increased to 80% of the W_max_. Participants were asked to cycle at a cadence of 60 revolutions per minute. Breath-by-breath measurements were collected of minute ventilation, breathing pattern, rate of oxygen consumption (VO_2_), and rate of expired carbon dioxide production (VCO_2_) (Medgraphics CardioO_2_; Medical Graphics Corporation). Measures of heart rate (HR) and SpO_2_ were continuously monitored and recorded using a 12-lead electrocardiogram and an ear probe attached to a pulse oximeter (Ohmeda Biox 3700e ear probe, Boulder, CO, USA). Blood pressure (BP) was measured automatically every 2 min (Tango M2; Suntech, Durham, NC, USA). Breathlessness and muscle fatigue were recorded each minute using a modified Borg scale [31]. Standardized encouragement was provided.

The ramp-base cycle ergometry test was performed using identical equipment and encouragement as described for the constant work rate test. To assess peak exercise capacity, participants completed 1 min of rest, 1 min of unloaded cycling, followed by a ‘continuous ramp’ protocol to symptom limitation. The magnitude of change in work rate was individualized based on the participant’s age and level of fitness, with the aim of achieving symptom limitation in 8 to 12 min [32].

### 5.2. Secondary Outcomes

#### Health-Related Quality of Life, Exercise Self-Efficacy, Feelings of Anxiety and Depression, and Enjoyment

Health-related quality of life was measured using two questionnaires: (i) the Cystic Fibrosis Questionnaire-Revised (CFQ-R) and (ii) the Alfred Wellness Score for CF (AweScore-CF) [33,34]. Exercise self-efficacy, that is, how confident a person feels towards completing exercise training, was measured using the Barriers Self-efficacy Scale (BARSE) [35]. Feelings of anxiety and depression were measured using the Hospital Anxiety and Depression Scale (HADS) [36]. Enjoyment was measured using the Physical Activity Enjoyment Scale (PACES) [37]. Further details of these questionnaires have been reported elsewhere [25].

## 6. Measurements Related to the Secondary Research Questions

### 6.1. Post-Exercise Muscle Soreness

Participants in the experimental group were asked if they experienced post-exercise quadriceps femoris muscle soreness whilst completing a ‘sit to stand’ 24 h following the first training session of each week. Those who reported experiencing post-exercise muscle soreness were asked to rate its severity using a Visual Analogue Scale (VAS) [38].

### 6.2. Tolerance

Participant adherence and completion data were recorded throughout the intervention period. Adverse events were monitored and recorded.

### 6.3. Debriefs

Participants allocated to the experimental group were invited to undertake an audio-recorded semi-structured interview (debrief) upon completion of the intervention period. Each interview was designed to take approximately 20 min. Open-ended questions were used to guide the sessions. Audio-recordings were transcribed verbatim to allow for thematic analysis.

### 6.4. Statistical Analyses

The sample size calculation has been published previously [25]. The results of this study were analyzed according to the intention-to-treat principle using STATA (version 15, StataCorp, College Station, TX, USA) and the analysis plan was developed in consultation with a biostatistician. Median and interquartile range was used to describe the data (unless stated). The magnitude of between-group change from baseline to follow-up was analyzed using rank-sum (Mann-Whitney U) tests and reported using *p*-values. Data obtained through audio-recorded HIIT sessions and debrief interviews were analyzed using Nvivo (version 12, QSR International Pty Ltd., Burlington, MA, USA).

## 7. Results

### Recruitment and Retention

The study flow diagram is presented in Figure 1. Fifteen participants were eligible and willing to take part in the study. Prior to randomization, one participant decided to withdraw and pursue an independent exercise program. Data collected during the first assessment session on this participant were excluded from any further analysis. The remaining 14 participants (median (IQR) age 31 (28, 35) years, FEV_1_ 61 (45, 80) % predicted) were randomized to the experimental (*n* = 7) or control (*n* = 7) groups. Participant characteristics are presented in Table 1.

## 8. Measures Related to the Primary Research Question

### 8.1. Primary Outcome

#### Endurance Exercise Capacity

Compared with the magnitude of improvement in T_lim_ (seconds) seen in the control group, greater change (increase) was seen in the experimental group (between-group difference *p* = 0.017). There were no other between-group differences (Table 2).

### 8.2. Secondary Outcomes

#### 8.2.1. Peak Exercise Capacity

Compared with the magnitude of change in W_max_ (Watts; W) seen in the control group, greater change (increase) in W_max_ was seen in the experimental group (between-group difference *p* = 0.017). There were no other between-group differences (Table 2).

#### 8.2.2. Health-Related Quality of Life

Compared with the magnitude of improvement in the physical function domain of the CFQ-R seen in the control group, greater change (improvement) was seen in the experimental group (between-group difference *p* = 0.03). There were no other between-group differences. Data for HRQoL are available in Table S2.

#### 8.2.3. Exercise Self-Efficacy, Feelings of Anxiety and Depression, Exercise Enjoyment, and Lung Function

There were no between-group differences in the magnitude of change for the BARSE, HADS, PACES, or lung function following the intervention period. Data for these outcomes are available in Tables S2 and S3.

## 9. Measurements Related to the Secondary Research Questions

### 9.1. Post Exercise Muscle Soreness

Four of the seven participants reported post-exercise muscle soreness on a single occasion each over the eight-week intervention period. The severity of these symptoms was mild (median (IQR) 8 mm (5, 8) out of 100 mm on a VAS).

### 9.2. Tolerance and Exercise Intensity

The median (IQR) percentage of sessions attended by participants in the experimental group was 93% (83, 95). Of the sessions attended, 100% of the participants were able to complete each session in its entirety. Reasons provided by participants who missed individual HIIT sessions included work commitments (*n* = 9), travel (*n* = 1) and family commitments (*n* = 2).

The intensity achieved during the final two weeks of the intervention period the HIIT was 111% (83, 132) of the baseline W_max_. No minor or major adverse events occurred.

### 9.3. Debrief Interviews

Four major themes surrounding facilitators to participating in the HIIT program were identified during the interviews. These themes were: (i) tolerability, (ii) commitment and flexibility, (iii) enjoyment, and (iv) the presence of a therapist during the HIIT sessions.

A number of actual and/or potential barriers to participating were identified during the interviews. These barriers fell into the following themes: (i) perceived or actual lack of internal motivation to undertake exercise, (ii) the travel time associated with session visits, (iii) life commitments, and (iv) the impact of the disease process. Verbatim examples from the debrief interview transcripts are listed in Table 3.

## 10. Discussion

This RCT demonstrated that eight weeks of low-volume HIIT produced a greater magnitude of change in T_lim_, W_max_, and self-reported physical function over and above the magnitude of change observed by continuing usual care (control group) in people with CF. The HIIT sessions were well tolerated, with only a few reports of mild-severity post-exercise muscle soreness, and high rates of attendance and completion among participants allocated to the experimental group. This RCT adds to the dearth of evidence for the use of HIIT in people with chronic respiratory disease, specifically CF [20].

Stability in median measures of exercise capacity in the control group, particularly T_lim_ and W_max_, increases our confidence in attributing the between-group difference to the HIIT program. The current study has demonstrated that T_lim_, which is traditionally used as a measure of submaximal endurance exercise capacity, is highly responsive in people with CF, and more responsive than measures obtained during a ramp-based cycle ergometry test. Although the use of the constant work rate cycle ergometry tests is novel in people with CF, results are likely to be more meaningful to clinicians and patients, because activities of daily living are usually undertaken at a submaximal exercise intensity. To this effect, clinicians could explain to patients that improving T_lim_ may translate to an improvement in their ability to perform daily activities for longer. While there were no between-group changes in the ‘gold standard’ measure of VO_2peak_ during the ramp-based cycle ergometry test, gains were seen in W_max_. Recent data from an international multicentre study demonstrated that W_max_ was a predictor of both survival and need for lung transplantation at 10-year follow-up in people with CF [8]. This means that improving W_max_ is an important treatment goal of exercise training and that improvements in W_max_ can be achieved with only 30 min of HIIT per week.

Although the minimal clinically important differences (MCID) for T_lim_ and W_max_ in people with CF are yet to be established, in people with COPD, the MCIDs were estimated to be 100 s and ~10 W [30], respectively. The magnitude of the between-group difference in favor of the experimental group in T_lim_ and W_max_ exceeded these thresholds, suggesting that these gains may be clinically important in people with CF. Nevertheless, our modest sample size precluded us from using parametric tests to explore between-group differences, and as such, we were unable to calculate the 95% confidence intervals and offer any precision around our estimate of these differences.

Excluding self-reported physical function (CFQ-R), the effects of HIIT on other components of HRQoL, and exercise self-efficacy, feelings of anxiety and depression and exercise enjoyment are uncertain. This may be related to the lack of CF-specific questionnaires to measure the majority of these outcomes with generic questionnaires likely to be less responsive to changes than disease-specific questionnaires. Furthermore, the modest sample size was likely to reduce our ability to detect small between-group difference in these generic outcomes.

Once participants agreed to participate in the study, attendance and completion rates were high. However, uptake into the study was low. This low uptake is likely due to the fact that participants were required to attend one of two facilities to complete the HIIT sessions (total of 22 sessions over 8 weeks). Indeed, the travel time associated with session visits and other life commitments were revealed as some of the main barriers to participation during debrief interviews. Given the facilitator themes of tolerability, flexibility, and exercise enjoyment, future studies should consider exploring the effect and uptake of the HIIT program provided in a home-based setting, with monitoring via telehealth. This may also facilitate long-term adherence to such a program.

One of the possible mechanisms that could have contributed to improvements in exercise capacity is improved respiratory muscle strength and function [3], which was not measured in this study. Future exercise training and HIIT studies should consider investigating the effects of these outcomes in people with CF.

## 11. Limitations

It is clear that recruiting participants posed a significant challenge, and the sample size calculated *a priori* was not met. As such, the results may not be generalizable to the wider population of people with CF. Due to the successful of recruitment of people with CF in previous non-exercise studies conducted within our group [39], it was presumed that the recruitment rate for this RCT would be higher. In retrospect, it would have been beneficial to undertake this as a feasibility study in the first instance. Despite the small sample size, improvements were demonstrated in the primary outcome of T_lim_, and secondary outcomes of W_max_ and self-reported physical function. In addition, participant commitment to the HIIT program was high once enrolled. In addition, no adverse events occurred. However, the small sample may have led to lack of between-group difference in other outcomes.

An intervention length of eight weeks was chosen by the research team, as this is the length of a ‘standard’ pulmonary rehabilitation program [40]. Given the lack of improvement in a number of secondary outcomes, an extension of three weeks (12 weeks in total) may have been beneficial [41].

## 12. Conclusions

The findings of this RCT indicate that 30 min of HIIT per week for eight weeks was superior to usual care for improving exercise capacity and self-reported physical function in this small group of people with CF. The improvement in the primary outcome of T_lim_ was approximately three times the projected MCID in people with chronic respiratory conditions [30]. Additionally, the HIIT program was well tolerated and accepted by participants. Future studies seeking to compare HIIT to usual care or to moderate intensity continuous exercise should consider interstate/international collaborative trials. Additionally, collection of biochemical markers of inflammation, body composition, and sleep would be useful to assess the systemic impact of HIIT in people with CF.

## Figures and Tables

**Table 1:** Participant characteristics.

Variable	Total Sample (*n* = 14)	Experimental Group (*n* = 7)	Control Group (*n* = 7)
Age (year)	31 (28, 35)	31 (29, 31)	31 (26, 39)
Height (cm)	176 (163, 182)	172 (163, 183)	179 (159, 182)
Sex (*n* female (%))	6 (43)	4 (57)	2 (29)
Weight (kg)	75 (64, 92)	75 (64, 101)	76 (63, 92)
BMI (kg·m^−2^)	23.92 (21.40, 30.25)	23.23 (21.40, 34.47)	24.60 (20.53, 28.49)
FEV_1_ (L)	2.20 (1.74, 3.40)	2.21 (1.90, 3.40)	1.81 (1.71, 3.58)
FEV_1_ (% predicted)	61 (45, 80)	66 (45, 83)	57 (39, 80)
FVC (L)	3.35 (3.07, 5.17)	3.30 (3.00, 5.60)	3.57 (3.07, 5.17)
FVC (% predicted)	82 (64, 95)	88 (62, 100)	79 (64, 92)
FEV_1_/FVC (%)	66 (58, 72)	67 (61, 72)	60 (54, 69)
**Comorbidity**	***n***	**%**	
Asthma	2	14	
CFRD	4	29	
CF-related liver disease	2	14	
Cholelithiasis	2	14	
Ileus	1	7	
OSA	1	7	
Osteopenia	3	21	
Pancreatic insufficiency	10	71	
Reflux	4	29	

Data are presented as median (IQR) unless otherwise stated. BMI: body mass index, CF: cystic fibrosis, CFRD: cystic fibrosis-related diabetes, FEV_1_: forced expiratory volume in 1 s, FVC: forced vital capacity, IQR: interquartile range, OSA: obstructive sleep apnea.

**Table 2:** Baseline and follow-up measures and comparison of between-group change in exercise capacity.

**Variable**	**Experimental Group (*n* = 5)**		**Control Group (*n* = 5)**		
**Ramp-Based Cycle Ergometry Test**	**Baseline**	**Follow-Up**	**Baseline**	**Follow-Up**	**Between-Group Difference**
VO_2peak_ (L·min^−1^)	2.25 (1.70, 2.79)	2.76 (2.12, 3.72)	2.25 (1.62, 2.31)	2.25 (1.53, 2.31)	*p* = 0.50
VO_2peak_ (mL·kg^−1^·min^−1^)	32.57 (22.60, 39.75)	40.75 (28.17, 42.96)	27.14 (21.70, 31.00)	27.14 (20.18, 30.23)	*p* = 0.20
VO_2peak_ (% predicted)	93 (75, 105)	90 (75, 114)	64 (62, 69)	64 (58, 69)	*p* = 0.16
VCO_2peak_ (L·min^−1^)	2.84 (2.13, 3.52)	3.20 (2.50, 4.42)	2.74 (2.23, 2.81)	2.20 (2.12, 2.62)	*p* = 0.27
W_max_ (Watts)	179 (122, 241)	250 (135, 253)	200 (161, 207)	202 (146, 203)	*p* = 0.017 **
W_max_ (% predicted)	90 (84, 138)	93 (90, 148)	82 (70, 85)	80 (68, 82)	*p* = 0.017 **
VE_peak_ (L·min^−1^)	85.44 (60.58, 107.00)	99.50 (74.94, 134.00)	61.37 (60.00, 71.10)	60.66 (56.73, 67.00)	*p* = 0.10
End-test breathlessness	7 (6, 8)	8 (7, 9)	5 (4, 8)	5 (4, 6)	*p* = 0.62
End-test leg muscle fatigue	9 (8, 9)	9 (7, 10)	9 (9, 9)	5 (5, 9)	*p* = 0.25
Nadir SpO_2_ (%)	93 (92, 94)	93 (91, 95)	93 (93, 94)	93 (89, 96)	*p* = 0.71
HR_max_ (bpm)	166 (152, 172)	172 (158, 173)	161 (159, 162)	159 (159, 159)	*p* = 0.17
HR_max_ (% predicted)	86 (81, 89)	86 (84, 91)	85 (84, 89)	85(83, 88)	*p* = 0.19
End-test RR (breaths·min^−1^)	44 (40, 51)	44 (42, 50)	32 (31, 43)	33 (30, 40)	*p* = 0.64
O_2_ pulse	14 (11, 16)	16 (11, 21)	14 (11, 15)	14 (11, 15)	*p* = 0.56
AT (% VO_2peak_)	45 (41, 57)	46 (43, 63)	28 (27, 32)	31 (29, 47)	*p* = 0.46
VO_2_/work slope (mL·min^−1^ W^−1^)	10.40 (9.00, 10.77)	10.39 (9.94, 11.49)	9.70 (9.40, 9.80)	9.72 (8.50, 9.80)	*p* = 0.58
**Variable**	**Experimental Group (*n* = 6)**		**Control Group (*n* = 5)**		
**Constant Work Rate Cycle Ergometry Test**	**Baseline**	**Follow-Up**	**Baseline**	**Follow-Up**	**Between-Group Difference**
T_lim_ (s)	276 (222, 360)	555 (317, 620)	248 (238, 262)	230 (228, 262)	*p* = 0.017 **
VO_2peak_ (L·min^−1^)	2.23 (1.60, 2.90)	2.21 (1.69, 2.98)	2.14 (1.43, 2.36)	2.08(1.57, 2.22)	*p* = 0.27
VO_2peak_ (mL·kg^−1^·min^−1^)	31.48 (21.55, 40.27)	31.8 (22.53, 40.68)	23.47 (20.47, 31.01)	23.47 (19.40, 29.64)	*p* = 0.27
VO_2peak_ (% predicted)	92 (67, 105)	95 (73, 112)	67 (58, 68)	62 (56, 64)	*p* = 0.20
VCO_2peak_ (L·min^−1^)	2.46 (1.89, 3.16)	2.39 (1.91, 2.88)	2.60 (2.35, 2.85)	2.29 (2.17, 2.67)	*p* = 1.00
VE_peak_ (L·min^−1^)	84.31 (55.91, 104.00)	84.22 (64.00, 107.97)	57.06 (55.61, 68.60)	57.36 (55.79, 67.00)	*p* = 0.86
End-test breathlessness	7 (5, 7)	7 (5, 9)	7 (7, 7)	7 (6, 7)	*p* = 0.36
End-test leg muscle fatigue	9 (7, 9)	8 (5, 10)	7 (5, 9)	5 (4, 9)	*p* = 0.92
Nadir SpO_2_ (%)	93 (90, 95)	94 (89, 95)	96 (95, 97)	95 (94, 96)	*p* = 0.13
HR_max_ (bpm)	163 (148, 169)	164 (158, 168)	156 (155, 162)	156 (155, 160)	*p* = 0.26
End-test RR (breaths·min^−1^)	44 (43, 44)	46 (45, 47)	30 (28, 32)	38 (33, 40)	*p* = 0.07

Data are presented as median (IQR). Between-group data were analyzed using rank-sum tests (reported as *p* values). ** Between-group difference in the magnitude of change from baseline to follow-up. Abbreviations: AT: anaerobic threshold, bpm: beats per minute, HR_max_: maximal heart rate, IQR: interquartile range, O_2_ pulse: oxygen pulse, RR: respiratory rate, SpO_2_: oxygen saturation, T_lim_: time to symptom limitation, VCO_2peak_: peak rate of carbon dioxide production, VE_peak_: peak minute ventilation, VO_2peak_: peak rate of oxygen uptake, W_max_: maximal work rate. End-test symptoms measured using the Borg scale (0 to 10). Predictive values from Jones et al. used for cycle ergometry tests.

**Table 3:** Note: P = participant number. (**a**) Debrief themes—facilitators; (**b**) Debrief Themes—Barriers.

(**a**)	
Tolerability	“The first couple times my legs were a bit sore, but it wasn’t really that much, it wasn’t unbearable, it wasn’t like it hurt to walk or anything like that” (P2) “I actually felt more energized afterwards” (P2) “It was certainly challenging, it wasn’t a walk in the park, but then the feeling I got after I had done a session was like an adrenaline rush… it was really motivating” (P8) “Most of the time it was my legs that got to me more than my breathing” (P8) “If I was having a bad day with my lungs, that was more effort (to attend), but I also know that when I felt that way we just adjusted the intensity that day accordingly, so I didn’t ever feel like I was going to ‘over-do’ it” (P8) “I was never sore, I never had leg pain or anything like that… I always felt good after, more energy and motivation” (P9) “I never had muscle soreness or anything like that, I knew I had done exercise, but that was it” (P11) “It wasn’t so hard that I couldn’t do it, but hard enough that it pushed me” (P14)
Commitment/ Flexibility	“It was easy to do because it was only 10 min, you only had to sweat it out for 10 min” (P2) “It was quite flexible with my work schedule” (P2) “The sessions weren’t ‘easy’ but the fact that it was a small amount of time really worked for my schedule” (P2) “It didn’t have a massive impact on my routine” (P8) “If I did a good session, I didn’t feel like I needed much else (additional exercise)” (P8) “The fact that the physio was so available (to supervise) all the time, to fit in with my schedule. I liked that” (P9) “The fact that it was quick and it was going to be over soon, it’s like, I can do this” (P9)
Enjoyment	“The actual sessions were challenging, but as much as they were challenging, I felt a real adrenaline rush after them, and it felt like a sense of achievement after each session, so yeah, really positive, I enjoyed it” (P8) “My experience overall was really positive, this was higher intensity exercise and a different style to how I would normally exercise. I really enjoyed that” (P8) “I was buzzing after doing the study, I even tried some interval running after the study finished with some friends” (P8) “I got into a routine, coming in here, I enjoyed it… it was an enjoyable experience” (P11) “You look forward it” (P9) “Short and sweet… I really enjoyed it!” (P9) “I really enjoyed it… I am sad that it is finishing, I probably will incorporate it into my routine going forward” (P11) “I would test myself, using the Watts on the bike, I could see the number, so I actually really enjoyed that, I would definitely incorporate it (the training) into my daily, weekly exercise routine, yeah, I enjoyed it” (P11) “I really enjoyed the structure of it (the program), having to be at a certain place at a certain time, it gave me focus” (P14) “I think because I enjoyed it, that was a real enabler (for me to continue)” (P14)
Therapist Presence	“Having the little cheer squad on the side really helps, because it is not something that I would probably do on my own. I think having the physio there to go ‘ok let’s get this done’ is much easier” (P2) “The supervision component was definitely motivating, I liked the structure” (P8) “The supervision component was really beneficial” (P8) “Having someone here, and knowing you’re only going to be exercising for a little bit makes it better” (P9) “I like to be pushed” (P9) “Having the intensity (Watts) and the number of bars (intervals) remaining on the screen, we also spoke about the heart rate; I liked having information and feedback because it helped me motivate and challenge myself for the next session, so yeah, I felt like I had good information from the physiotherapist throughout” (P11) “Knowing there was going to be someone supervising, you couldn’t just miss a session because you’d be letting someone down. I think I enjoyed that, it was a real enabler” (P11) “Say, for example I had the equipment at home or at the local gym, and you said ‘go three times per week’, I probably wouldn’t have felt as motivated, but having someone to encourage along the way, it is something different to what I have had before” (P14)
(**b**)	
Internal Motivation	“If I skipped one session, it was tougher the next time” (P2) “Motivation for me is a tough one, like I said, I didn’t like, I still don’t like exercise, and it is not something I will ever be comfortable with, yep, not my thing… that was probably my biggest struggle, the motivation to do it, and I found if I missed a session, it was even harder the next time” (P2) “I reverted back to my old ways after finishing the program, but I don’t have the equipment (bike) at home, so I wasn’t going to continue anyway…” (P2) “I am not one for exercise… I kind of feel like I am allergic to it!” (P2) “I don’t know what to do if I am by myself, what (exercise) is going to be effective and what’s not” (P9) “I think if I am going to do it (the HIIT) on my own, I need to find the right gym… because I need the motivation” (P9) “Probably just not having the motivation (is the main barrier to exercise). When someone is there with a plan… it is probably just my own laziness when it comes down to it, I need to stop being lazy” (P9) “I haven’t done any exercise since (completing the program)” (P9) “Once you make a commitment to someone, you can’t change it, so it was like ‘I have to go’ (to the HIIT sessions), whereas now I will be on my own, it could be easier to tell myself I don’t feel like it today” (P11) “If I hadn’t enjoyed the sessions, that would have been a barrier” (P11) “Some days I just don’t have the energy (to exercise)… it’s the motivation, definitely” (P14)
Travel/ Location	“The main thing was finding parking” (P2) “Being at home would be better” (P2) “I was lucky that I worked down the road, so if it was any further, that would have been a barrier” (P8) “I don’t have any (barriers). It’s easy for me… I live close and parking wasn’t an issue” (P11) “I work close by and live close by, so it was easy. Whereas if I perhaps lived or worked further away, it might have been more effort to get there” (P14)
Life/Other Commitments	“Having CF I am pretty busy, and I’ve got a child, and a full-time job, I work long hours, so it is important to me for it (exercise) to be flexible” (P2) “Mainly just work (was a barrier)… having to go around work” (P9) “If the sessions were longer, that could have been a barrier… trying to fit it in with everything else” (P14)
Disease	“It was tough during the time when I wasn’t well, but I was still able to do it (the HIIT)” (P2) “If I was having a bad day with my lungs, that was more effort (to attend), but I also know that when I felt that way we just adjusted the intensity that day accordingly, so I didn’t ever feel like I was going to ‘over-do’ it” (P8) “I guess if you are feeling sick it is obviously harder to exercise” (P9) “The better I am feeling, the more likely I am to go (to exercise)” (P14) “When you feel unwell, and then you have all your medication and you have to try and schedule exercise in, it’s just hard” (P14)

## Supplementary Materials

The following are available online at https://www.mdpi.com/2077-0383/9/10/3098/s1, Table S1: Behavior change techniques within high intensity interval training intervention, Table S2: Baseline and follow-up measures and comparison of between-group change in health-related quality of life, exercise self-efficacy, feelings of anxiety and depression, and exercise enjoyment, Table S3: Baseline and follow-up measures and comparison of between-group change in lung function.

## Abbreviations

ANZCTR, Australian New Zealand Clinical Trials Registry; AweScore-CF, Alfred wellness score for cystic fibrosis; BARES, Barriers self-efficacy scale; BMI, Body mass index; BP, Blood pressure; CF, Cystic fibrosis; CFQ-R, Cystic fibrosis questionnaire revised; CONSORT, Consolidated Standards of Reporting Trials; COPD, Chronic obstructive pulmonary disease; FEV1, Forced expiratory volume in one second; HADS, Hospital anxiety and depression scale; HIIT, High intensity interval training; HR, Heart rate; HRQoL, Health-related quality of life; MCID, Minimal clinically important difference; PACES, Physical activity enjoyment scale; PCH, Perth Children’s Hospital; PR, Pulse rate; RCT, Randomized controlled trial; SCGH, Sir Charles Gairdner Hospital; SpO2, Oxygen saturation measured using a pulse oximeter; Tlim, Time to symptom limitation; VAS, Visual Analogue Scale; VCO2, Rate of expired carbon dioxide production; VO2, Rate of oxygen consumption; VO2peak, Peak rate of oxygen uptake; Wmax, Maximal work rate.

## References

[1] Troosters T., Langer D., Vrijsen B., Segers J., Wouters K., Janssens W., Gosselink R., Decramer M., Dupont L. (2009). Skeletal muscle weakness, exercise tolerance and physical activity in adults with cystic fibrosis. Eur. Respir. J..

[2] Boucher R.C. (2004). New concepts of pathologenesis of cystic fibrosis lung disease. Eur. Respir. J..

[3] Dassios T. (2015). Determinants of respiratory pump function in patients with cystic fibrosis. Paediatr. Respir. Rev..

[4] De Jong W., Kaptein A.A., Van Der Schans C.P., Mannes G.P.M., Van Aalderen W.M.C., Grevink R.G., Koëter G.H. (1997). Quality of life in patients with cystic fibrosis. Pediatr. Pulmonol..

[5] Orenstein D.M., Nixon P.A., Ross E.A., Kaplan R.M. (1989). The quality of well-being in cystic fibrosis. Chest.

[6] Nixon P.A., Orenstein D.M., Kelsey S.F., Doershuk C.F. (1992). The prognostic value of exercise testing in patients with cystic fibrosis. N. Engl. J. Med..

[7] Pianosi P., Leblanc J., Almudevar A. (2005). Peak oxygen uptake and mortality in children with cystic fibrosis. Thorax.

[8] Hebestreit H., Hulzebos E.H., Schneiderman J.E., Karila C., Boas S.R., Kriemler S., Dwyer T., Sahlberg M., Urquhart D.S., Lands L.C. (2018). Cardiopulmonary exercise testing provides additional prognostic information in cystic fibrosis. Am. J. Respir. Crit. Care Med..

[9] Radtke T., Nevitt S.J., Hebestreit H., Kriemler S. (2015). Physical exercise training for cystic fibrosis. Cochrane Database Syst. Rev..

[10] Schneiderman J.E., Wilkes D.L., Atenafu E.G., Nguyen T., Wells G.D., Alarie N., Tullis E., Lands L.C., Coates A.L., Corey M. (2013). Longitudinal relationship between physical activity and lung health in patients with cystic fibrosis. Eur. Respir. J..

[11] Klijn P.H., Oudshoorn A., van der Ent C.K., van der Net J., Kimpen J.L., Helders P.J. (2004). Effects of anaerobic training in children with cystic fibrosis: A randomized controlled study. Chest.

[12] Selvadurai H.C., Blimkie C.J., Meyers N., Mellis C.M., Cooper P.J., Van Asperen P.P. (2002). Randomized controlled study of in-hospital exercise training programs in children with cystic fibrosis. Pediatr. Pulmonol..

[13] Moorcroft A.J., Dodd M.E., Morris J., Webb A.K. (2004). Individualised unsupervised exercise training in adults with cystic fibrosis: A 1 year randomised controlled trial. Thorax.

[14] Button B.M., Wilson C., Dentice R., Cox N.S., Middleton A., Tannenbaum E., Bishop J., Cobb R., Burton K., Wood M. (2016). Physiotherapy for cystic fibrosis in Australia and New Zealand: A clinical practice guideline. Respirology.

[15] White D., Stiller K., Haensel N. (2007). Adherence of adult cystic fibrosis patients with airway clearance and exercise regimens. J. Cyst. Fibros..

[16] Gruber W., Orenstein D.M., Braumann K.M., Beneke R. (2014). Interval exercise training in cystic fibrosis- Effects on exercise capacity in severely affected adults. J. Cyst. Fibros..

[17] Gibala M.J., Little J., MacDonald M.J., Hawley J.A. (2012). Physiological adaptations to low-volume, high-intensity interval training in health and disease. J. Physiol..

[18] Helgerud J., Høydal K., Wang E., Karlsen T., Berg P., Bjerkaas M., Simonsen T., Helgesen C., Hjorth N., Bach R. (2007). Aerobic high-intensity intervals improve VO_2_max more than moderate training. Med. Sci. Sports Exerc..

[19] Weston M., Taylor K.L., Batterham A.M., Hopkins W.G. (2014). Effects of low-volume high-intensity interval training (HIT) on fitness in adults: A meta-analysis of controlled and non-controlled trials. Sports Med..

[20] Sawyer A., Cavalheri V., Hill K. (2020). Effects of high intensity interval training on exercise capacity in people with chronic pulmonary conditions: A narrative review. BMC Sports Sci. Med. Rehabil..

[21] Arnardóttir H.R., Boman G., Larsson K., Hendenström H., Emtner M. (2007). Interval training compared with continuous training in patients with COPD. Respir. Med..

[22] Mador M.J., Krawza M., Alhjhusian A., Khan A.I., Shaffer M., Kufel T.J. (2009). Interval training versus continuous training in patients with chronic obstructive pulmonary disease. Cardiopulm. Rehabil. Prev..

[23] Gillen J.B., Martin B.J., MacInnis M.J., Skelly L.E., Tarnopolsky M.A., Gibala M.J. (2016). Twelve weeks of sprint interval training improves indices of cardiometabolic health similar to traditional endurance training despite a five-fold lower exercise volume and time commitment. PLoS ONE.

[24] Puhan M.A., Büsching G., Schünemann H.J., VanOort E., Zaugg C., Frey M. (2006). Interval versus continuous high-intensity exercise in chronic obstructive pulmonary disease: A randomized trial. Ann. Intern. Med..

[25] Sawyer A., Cavalheri V., Jenkins S., Wood J., Cecins N., Singh B., Hill K. (2018). Effects of high intensity interval training on exercise capacity in people with cystic fibrosis: Study protocol for a randomised controlled trial. BMC Sports Sci. Med. Rehabil..

[26] Hoffmann T.C., Glasziou P.P., Boutron I., Milne R., Perera R., Moher D., Altman D.G., Barbour V., Macdonald H., Johnston M. (2014). Better reporting of interventions: Template for intervention description and replication (TIDieR) checklist and guide. BMJ.

[27] Slade S.C., Dionne C.E., Underwood M., Buchbinder R. (2014). Standardised method for reporting exercise programmes: Protocol for a modified Delphi study. BMJ Open.

[28] Slade S.C., Dionne C.E., Underwood M., Buchbinder R., Beck B., Bennell K., Brosseau L., Costa L., Cramp F., Cup E. (2016). Consensus on exercise reporting template (CERT): Modified delphi study. Phys. Ther..

[29] Goss C.H., Burns J.L. (2007). Exacerbations in cystic fibrosis·1: Epidemiology and pathogenesis. Thorax.

[30] Puente-Maestu L., Palange P., Casaburi R., Laveneziana P., Maltais F., Neder J.A., O’Donnell D.E., Onorati P., Porszasz J., Rabinovich R. (2016). Use of exercise testing in the evaluation of interventional efficacy: An official ERS statement. Eur. Respir. J..

[31] Borg G. (1982). Psychophysical bases of perceived exertion. Med. Sci. Sports Exerc..

[32] American Thoracic Society, American College of Chest Physicians (2003). ATS/ACCP statement on cardiopulmonary exercise testing. Am. J. Respir. Crit. Care Med..

[33] Button B.M., Gufler A., Clark D., Mitchell L., Wilson J.W. (2014). The development of a new quick/easy CF wellness score (Alfred Wellness Score for CF, “AweScore-CF”) to improve delivery of clinical care in the outpatient and inpatient settings suggests patients acclimatise to low lung function. J. Cyst. Fibros..

[34] Quittner A.L., Buu A., Messer M.A., Modi A.C., Watrous M. (2005). Development and validation of the cystic fibrosis questionnaire in the United States: A health-related quality-of-life measure for cystic fibrosis. Chest.

[35] McAuley E. (1992). The role of efficacy cognitions in the prediction of exercise behavior in middle-aged adults. J. Behav. Med..

[36] Snaith R.P. (2003). The Hospital Anxiety and Depression Scale. Health Qual. Life Outcomes.

[37] Kendzierski D., DeCarlo K.J. (1991). Physical activity enjoyment scale: Two validation studies. J. Sport Exerc. Psychol..

[38] Bijur P.E., Silver W., Gallagher E.J. (2001). Reliability of the visual analog scale for measurement of acute pain. Acad. Emerg. Med..

[39] Wood J., Jenkins S., Putrino D., Mulrennan S., Morey S., Cecins N., Bear N., Hill K. (2020). A smartphone application for reporting symptoms in adults with cystic fibrosis improves the detection of exacerbations: Results of a randomised controlled trial. J. Cyst. Fibros..

[40] Jenkins S., Hill K., Cecins N.M. (2010). State of the art: How to set up a pulmonary rehabilitation program. Respirology.

[41] Orenstein D.M., Franklin B.A., Doershuk C.F., Hellerstein H.K., Germann K.J., Horowitz J.G., Stern R.C. (1981). Exercise conditioning and cardiopulmonary fitness in cystic fibrosis: The effects of a three-month supervised running program. Chest.

