# Supplementary material for: High-Intensity Interval Training Is Effective at Increasing Exercise Endurance Capacity and Is Well Tolerated by Adults with Cystic Fibrosis
Source: J Clin Med. 2020 Sep 25;9(10):3098. doi: 10.3390/jcm9103098 (PMC7601335; doi:10.3390/jcm9103098)
Supplement: Supplementary file 1 [file jcm-09-03098-s001.pdf]

The HIIT sessions were audio recorded over the eight-week intervention period. The audio-recordings were transcribed verbatim and used to assist in the analysis of the program using the BCT Taxonomy v1 [1].

- **Target population:** Experimental group (adults with cystic fibrosis (CF) who were allocated to undertake eight weeks of high intensity interval training (HIIT))
- **Target behavior:** Optimize participation in HIIT program: (i) attendance at each individual HIIT session, (ii) completion of each individual HIIT session, and (iii) completion of at least 70% of the total sessions.
- **Target outcome**: Increase exercise capacity as determined by time to symptom limitation (primary outcome) during a constant work rate cycle ergometry test (at program completion). 'Proxy' measures of exercise capacity or 'indices' of intensity were taken from the work rate achieved during HIIT sessions, as well as symptom scores and heart rate readings.

Fifteen out of a possible 93 BCTs [1] were included within the HIIT intervention. Three BCTs were present on more than one occasion. Of the 18 occasions that a BCT was employed overall, the BCT could be mapped with a high level of confidence on 15 occasions.

Table S1. Behavior change techniques within high intensity interval training intervention.

Description of intervention component	Behavior change techniques identified	Confidence
When in attendance at the HIIT sessions, participants were asked to increase the intensity of exercise each week depending on what they were able to tolerate (based on their perceived level of leg muscle fatigue and breathlessness, as a proxy measure of increasing exercise capacity). Sessions commenced at 60% of the W_{max} in week 1, with the aim of achieving 80% of the W_{max} by the end of week 2 (lead-in phase), and as symptoms permit from week 3 to week 8 of the program.	Goal setting (behavior)	2
	Graded tasks	2
Participants were provided with detailed instructions to attend the physiotherapy department (at their preferred hospital location) two, and then three, times per week for 8 weeks to undertake each HIIT session. Information was also provided on parking options at the hospital location via a map (as required). The same physiotherapist was present for all HIIT sessions throughout the program. The participants were able to contact the physiotherapist by phone or email to discuss booking sessions, various aspects of the program and any issues encountered.	Action planning	2
	Instruction on how to perform the behavior	2
	Social support (unspecified)	2
Participants were provided with verbal and written (an information sheet) instructions on post-exercise muscle soreness and how to manage potential symptoms prior to undertaking their first HIIT session. The verbal instructions were reiterated weekly and participants were advised to inform the physiotherapist of any delayed symptoms related to the HIIT.	Information about health consequences	2
Discussion occurred with participants prior to commencing each HIIT session regarding the previous intensity achieved (based on work rate, symptoms and heart rate) as well as the goal for this session. At the completion of each session, further discussion of the markers occurred in order to reflect on the intensity achieved during the session.	Review behavior goal(s)	2
	Feedback on outcome(s) of behavior	2
The intensity of exercise (in Watts, as a percentage of the participant's baseline level of fitness), and achievements of other participants (who had already completed the intervention period) were discussed with current participants to reflect on the progress they had made, in comparison to the progress of others that had been made at similar time points.	Social comparison	1

Participants were provided with verbal (by the physiotherapist) and visual (Watt display on the computer on the cycle ergometer) cues regarding how far they were through each interval, the intensity achieved during the interval and the time left for HIIT session. Observations (HR, SpO_2 and Borg scores, and Watts on the cycle ergometer) were monitored by the physiotherapist throughout each HIIT session and verbal feedback was provided by the physiotherapist to reassure the participant at regular time intervals on how they were responding to the session.	Biofeedback<br>Feedback on behavior	2<br>2
In order to convey the physiotherapist's confidence in the participant being able to achieve a specific intensity of exercise, participants were reminded about the intensity they were able to achieve in previous HIIT sessions, despite uncomfortable symptoms that may have been associated with the exercise. In addition, the physiotherapist provided positive reinforcement and encouragement when a particular intensity was achieved (i.e. based on Watts) despite the difficulty in achieving the behavior (i.e., onset of leg muscle fatigue and breathlessness).	Social reward<br>Verbal persuasion about capability<br>Focus on past success	2<br>1<br>2
The participants were advised that to have 'completed' the intervention, they were required to attend at least 70% of sessions. The session count was discussed with the participant weekly in order to plan for the remaining sessions/weeks and set goals for the exercise intensity to aim for in the remaining weeks.	Goal setting (behavior)<br>Review behavior goal(s)<br>Feedback on behavior	2<br>2<br>2
If a participant missed a session(s), they were permitted to extend the program by a maximum of 2 weeks. This was outlined to the participant at the start of the program, and if they had to cancel a session for any reason (life commitments, being medically unwell etc.)	Restructuring the physical environment	1

Abbreviations: HR: heart rate, SpO_2: oxygen saturation, W_{max}: maximal work rate. A '2' in the confidence column of the table indicates that the authors confident 'beyond reasonable doubt' that the technique was present. A '1' in the confidence column indicates that the technique was present 'in all probability'.

Reference:

1. Michie, S.; Richardson, M.; Johnston, M.; Abraham, C.; Francis, J.; Hardeman, W.; Eccles, M.P.; Cane, J.; Wood, C.E. The behavior change technique taxonomy (v1) of 93 hierarchically clustered techniques: building an international consensus for the reporting of behavior change interventions. *Ann Behav Med.* **2013**, *46*, 81–95.

Table S2. Baseline and follow-up measures and comparison of between-group change in health-related quality of life, exercise self-efficacy, feelings of anxiety and depression, and exercise enjoyment.

Variable	Experimental Group (n = 7)		Control Group (n = 7)		
	Baseline	Follow-Up	Baseline	Follow-Up	Between-Group Difference
**CFQ-R (total)**	72 (65, 83)	81 (69, 86)	72 (68, 85)	78 (66, 81)	p = 0.66
Physical function	75 (54, 92)	88 (63, 100)	92 (50, 92)	79 (50, 96)	p = 0.033 **
Vitality	50 (42, 75)	58 (33, 67)	50 (42, 75)	58 (42, 67)	p = 0.95
Emotional function	80 (73, 100)	87 (67, 100)	87 (73, 93)	87 (67, 87)	p = 0.44
Eating disturbance	100 (100, 100)	100 (89, 100)	100 (89, 100)	100 (89, 100)	p = 0.62
Treatment burden	67 (11, 78)	56 (22, 67)	56 (44, 67)	56 (44, 67)	p = 0.95
Health perception	67 (44, 89)	78 (56, 89)	67 (44, 78)	56 (44, 67)	p = 0.14
Social function	72 (61, 83)	72 (56, 83)	72 (67, 78)	72 (67, 83)	p = 0.25
Body image	78 (67, 89)	67 (56, 89)	56 (56, 100)	67 (56, 100)	p = 0.22

Role limitations	92 (67, 100)	92 (83, 100)	83 (75, 92)	83 (75, 92)	$p = 0.56$
Weight problems	100 (33, 100)	100 (33, 100)	100 (67, 100)	100 (67, 100)	$p = 0.79$
Respiratory symptoms	72 (50, 72)	72 (44, 89)	72 (56, 89)	67 (56, 83)	$p = 0.18$
Digestive symptoms	100 (89, 100)	89 (78, 100)	78 (78, 100)	78 (78, 89)	$p = 0.30$
**AweScore-CF (total)**	65 (50, 66)	62 (55, 67)	63 (48, 74)	65 (51, 69)	$p = 0.85$
**BARSE (total)**	59 (47, 78)	55 (35, 68)	48 (31, 64)	37 (34, 67)	$p = 0.90$
**HADS (total)**	8 (4, 14)	8 (3, 15)	4 (3, 8)	7 (3, 8)	$p = 0.95$
Anxiety	5 (3, 10)	6 (3, 9)	3 (3, 6)	4 (1, 6)	$p = 0.61$
Depression	3 (2, 4)	2 (1, 4)	1 (0, 1)	2 (1, 2)	$p = 0.32$
**PACES (total)**	38 (18, 44)	44 (24, 51)	32 (25, 49)	36 (17, 45)	$p = 0.12$

Data are presented as median (IQR) unless otherwise stated. Between-group data are analysed using rank-sum tests (reported as *p* value). ** Between-group difference in the magnitude of change from baseline to follow-up. Abbreviations: CFQ-R: Cystic Fibrosis Questionnaire-Revised, HADS: Hospital Anxiety and Depression Scale, PACES: Physical Activity Enjoyment Scale.

Table S3. Baseline and follow-up measures and comparison of between-group change in lung function.

	**Experimental Group ($n = 7$)**		**Control Group ($n = 7$)**		
	**Baseline**	**Follow-Up**	**Baseline**	**Follow-Up**	**Between-Group Difference**
FEV_1 (L)	2.2 (1.9, 3.4)	2.2 (1.9, 3.4)	1.8 (1.7, 3.6)	1.8 (1.5, 3.5)	$p = 1.00$
FEV_1 (% predicted)	66 (45, 83)	66 (53, 81)	57 (39, 80)	58 (37, 78)	$p = 0.95$
FVC (L)	3.3 (3.0, 5.6)	3.2 (2.5, 5.7)	3.6 (3.1, 5.2)	3.3 (3.1, 5.0)	$p = 1.00$
FVC (% predicted)	88 (62, 100)	83 (60, 98)	79 (64, 92)	68 (59, 92)	$p = 0.18$
FEV_1/FVC	67 (61, 72)	70 (64, 75)	60 (54, 69)	69 (51, 77)	$p = 0.48$

Data are presented as median (IQR) unless otherwise stated. Between-group data are analysed using rank-sum tests (reported as *p* value). Between-group difference in the magnitude of change from baseline to follow-up. Abbreviations: FEV_1: forced expiratory volume in 1 second, FVC: forced vital capacity, IQR: interquartile range. Global Lung Index Initiative lung function reference values used [386]. Note: The FEV_1/FVC ratio increased from 60 to 69% in the control group following the intervention period. This change appears to reflect a reduction in the median FVC % predicted in both groups.
